# Women’s status, breastfeeding support, and breastfeeding practices in the United States

**DOI:** 10.1371/journal.pone.0275021

**Published:** 2022-09-28

**Authors:** Jennifer Yourkavitch, Paige Hall Smith

**Affiliations:** 1 Department of Maternal and Child Health, University of North Carolina, Chapel Hill, North Carolina, United States of America; 2 Department of Public Health Education, University of North Carolina Greensboro, Greensboro, North Carolina, United States of America; Christiana Care/University of Delaware, UNITED STATES

## Abstract

The objective of this study is to examine associations between state-level breastfeeding support and breastfeeding practices, controlling for women’s status, in the U.S. We used publicly available data on state-level breastfeeding practices and support (international board-certified lactation consultants (IBCLC), births in Baby-Friendly hospitals, and La Leche League Leaders) for births in 2015 from the CDC Breastfeeding Report Card (2018) and other CDC reported data, and indicators of women’s status from the Institute for Women’s Policy Research reports (2015). We conducted an ecological study to estimate incidence rate ratios of exclusive breastfeeding at six months and breastfeeding at 12 months with breastfeeding supports using bivariate and multivariable Poisson regression. Political participation, poverty, and employment and earnings were associated with breastfeeding practices, as was each breastfeeding support in bivariate analyses. After controlling for women’s status, only IBCLCs were positively associated with rates of exclusive breastfeeding at 6 months and continued breastfeeding at 12 months. For every additional IBCLC per 1000 live births, the rate of exclusive breastfeeding at 6 months increased by 5 percent (95% CI 1.03, 1.07) and the rate of breastfeeding at 12 months increased by 4 percent (95% CI 1.02, 1.06). Political participation, poverty, and employment and earnings were associated with breastfeeding practices, indicating a relationship between women’s political and economic status and their breastfeeding practices in the U.S. Given the influence of women’s status, increasing the number of IBCLCs may improve breastfeeding practices.

## Introduction

Women’s status, the structural social, economic and political disadvantages and advantages experienced by women due to gender norms, and gender equality, the relative difference in status between men and women, have long been associated with maternal and child health around the world [1]. Some state-level studies investigating the impact of women’s status on health outcomes in the U.S. use measures based on domains defined and measured by the Institute for Women’s Policy Research (IWPR), which include: political participation, work and family, employment and earnings, poverty, and reproductive rights, among others [2].

### The status of women and health research

Over the last few decades studies that use these data to investigate relationships between women’s status and health outcomes have found positive associations with maternal and child, and even men’s, health outcomes, although the character of the relationships have differed. A 2006 study using the IWPR conceptualization found that child and adolescent health outcomes at the state level (e.g., infant mortality, percent low birth weight, and teen pregnancy) were all inversely associated with some or all of the measures of women’s status; after adjusting for state level measures of income inequality and race these outcomes remained significantly inversely associated with one or more of the measures of women’s status [3]. In terms of adult health, Roberts found that, with the exception of reproductive rights, higher gender equality or women’s status is associated with less risky drinking for both women and men [4]. Prior studies recommend that additional research needs to incorporate indicators of other resources or supports that might shape the relationships between the status of women and health.

### The status of women and breastfeeding

The American Academy of Pediatrics recommends exclusive breastfeeding for the first six months of life and continued breastfeeding for one or more years [5], and research enumerates many benefits for children’s health in addition to benefits for women’s health, including reduced risk of breast and ovarian cancers, and metabolic disease [6]. However, only 25% of infants born in 2015 were exclusively breastfed for six months and 36% were breastfed for one year [7]. Most women in the US are aware of the benefits of breastfeeding and over 80% of infants are breastfed at some point [7]. However, research suggests that women lack the resources and support they need to navigate the difficulties they encounter as they seek to work, live, socialize, and play in public spaces [8]. Given this lack of support, some scholars have seen breastfeeding promotion as antagonist to efforts to improve the status of women [9] due to its association with women’s roles as unpaid caregivers, with loss of autonomy that could come from using their bodies to feed babies, and with spending significant time in their role as a mother.

In addition, it might be reasonably hypothesized that women would choose to feed their infants with artificial milk substitutes rather than their breasts as their socio-economic and employment options and status increased, and there is some evidence that this has occurred. A series of studies using data from the Demographic and Health Surveys (DHS) and World Fertility Survey, from the 1970s and 1980s across many low- and middle-income countries revealed declines in breastfeeding as women achieved gains in education and income and changes from rural to urban residence [10, 11]. Of note is the fact that these same demographic changes, particularly increasing urbanization and female education, generally have led to improvements in other indicators of maternal and child health [12].

However, a study a decade later, using DHS data from 23 low- and middle-income countries between the mid- l980s and mid 1990s, found that the opposite pattern was occurring. Perez-Escamilla observed that while breastfeeding data from the previous decade would lead to the hypothesis that with further urbanization and improvements in women’s education that breastfeeding rates would decrease, data suggested just the opposite. Breastfeeding was decreasing among women without formal education while it was increasing among women with at least secondary education [13].

This pattern is what we observe in the U.S. today. Data at the individual level reveal a range of social vulnerabilities that lead to reduced breastfeeding including: race, poverty, receiving Special Supplemental Nutrition Program for Women, Infants, and Children benefits, being unmarried, having less education, being younger, and having difficulties navigating breastfeeding and employment [14, 15].

Smith used the IWPR domains to examine the association of women’s status with breastfeeding initiation, duration and exclusivity [16]. This study, which combined women’s status indicators in one global measure due to high inter-item correlations, found that states where women’s global status was higher also had higher proportions of breastfeeding initiation, any breastfeeding at six and 12 months, and exclusive breastfeeding at three and six months. Smith concluded that higher status may make it easier for women to integrate their productive and reproductive lives; however, these findings also indicated that women who live in states where they have less economic, social, and political status may find it more difficult to reconcile the different roles and responsibilities in their lives [16]. This study did not consider how macro-level indicators of clinical resources and support may have contributed to breastfeeding outcomes.

### Professional and lay support for breastfeeding

Over the decades, as women’s status in the U.S. has risen, so has governmental, health care, workplace, and social support for breastfeeding [17]. As part of their annual national “breastfeeding report card” the Centers for Disease Control and Prevention (CDC) identifies multiple indicators of breastfeeding support at the state-level, including birth facility practices and professional breastfeeding support (international board-certified lactation consultants (IBCLC)), and the website includes an indicator of lay (La Leche League) breastfeeding support. A review of breastfeeding support in the hospital through the Baby-Friendly Hospital Initiative found an association between the program and breastfeeding practices [18], although a study of state-level breastfeeding outcomes did not find an association [19]. Reviews of professional and lay breastfeeding support found a positive effect on breastfeeding practices, including extending duration [20, 21], and some postulate that support may have a greater influence than socio-economic status on breastfeeding practices [22]. IBCLC is a professional certification attained after completing more than one thousand hours of academic study and mentored practice, and passing a standardized exam.

### Purpose of this study

We sought to examine associations between women’s status and breastfeeding in the United States, and to investigate how women’s status may influence the effectiveness of three different indicators of state-level breastfeeding resources and support: breastfeeding friendly hospitals; professional breastfeeding support; and lay breastfeeding support. Our study, unlike several prior studies using the IWPR conceptualization of women’s status, also incorporated a fifth dimension of women’s status that is now available in the IWPR database: work and family, which includes data related to paid leave, child care indicators, and the gender gap in parents’ labor force participation, among others [2].

## Methods

### Data

All data are state-level indicators from publicly available databases. No human subjects were involved in this data analysis. We included two state-level outcome indicators for breastfeeding practices: exclusive breastfeeding at 6 months and continued breastfeeding at 12 months. These values were collected for infants born in 2015 from the CDC database. Indicators of state-level breastfeeding support (the exposure) for 2015, reported on CDC’s website [7] included: the number of IBCLCs per 1000 births; the number of La Leche League (LLL) leaders per 1000 births; and, the percentage of births occurring in hospitals with the Baby-Friendly Hospital (BFH) designation.

We created a dataset of indicators for 50 states and the District of Columbia from two data sources. First, we assembled the values of five state-level indices of women’s status from 2013 compiled by the IWPR including: political participation, employment and earnings, work and family, reproductive rights, and poverty, which are defined elsewhere [2]. In brief, the indices combine several relevant indicators within each theme to provide a composite measure. We used the IWPR construction of indices except for “poverty” for which we used a single indicator instead—the proportion of women living in poverty—because that is the main issue within that domain that we wanted to examine. We did not use the IWPR health and wellness index because several indicators, including chronic disease and mental health, may have a bi-directional relationship with breastfeeding, which would complicate model interpretation. The status of women indices were used to control for the effects of women’s status on the association between breastfeeding support and breastfeeding practices at state level. We believe that women’s status, as reported in 2013, influenced women’s lives to a similar extent in 2015 as they did at the time of data collection.

### Analysis

First, we examined the distribution of the exposure and outcome variables and women’s status indices for all states. Second, we examined correlations among women’s status variables. Next, we estimated incidence rate ratios to examine associations of state-level breastfeeding support and women’s status with breastfeeding practices using bivariate Poisson regression with robust standard errors using the GENMOD procedure within SAS (v. 9.4, SAS Institute, Cary, NC). Finally, we used multivariable Poisson regression to estimate incidence rate ratios for the association of breastfeeding support with breastfeeding practices, controlling for women’s status. The multivariable models also controlled for the percentages of Black and Hispanic populations, following methods reported in Koenen et al. [3, 23].

## Results

**Fig 1:**
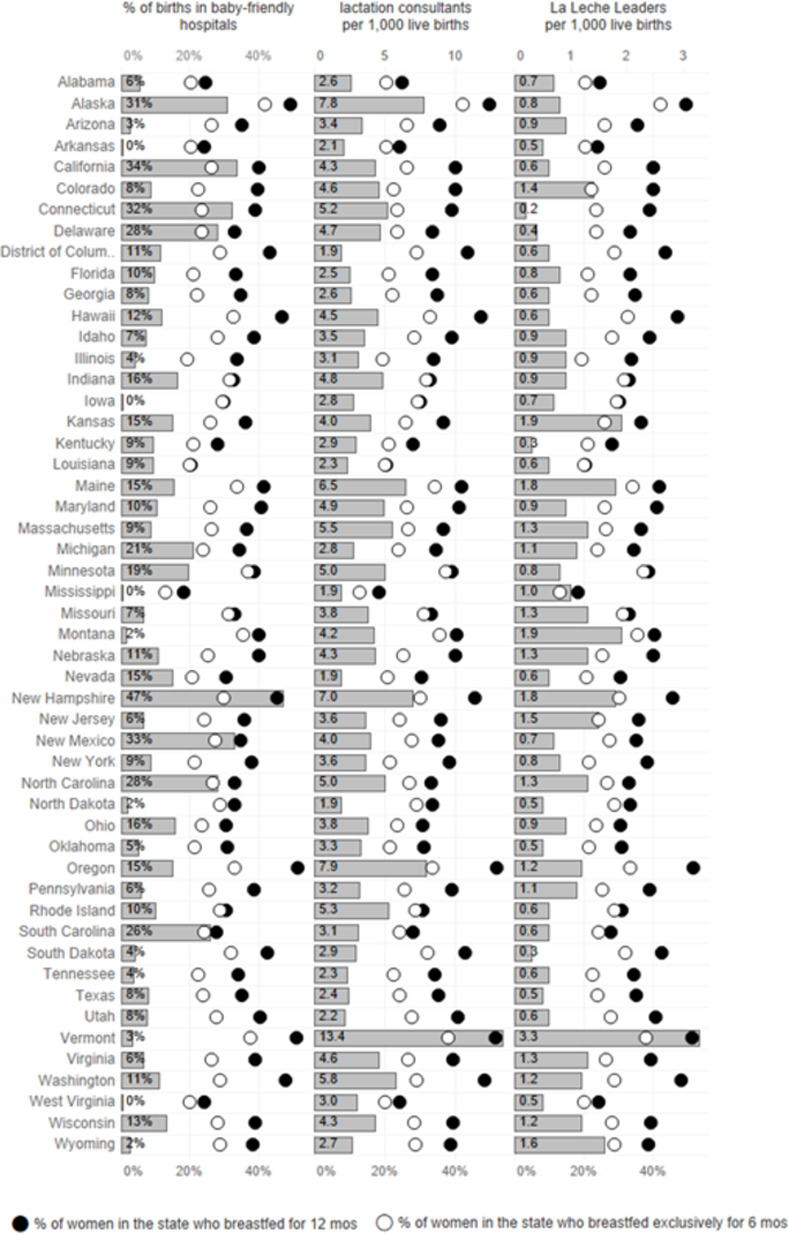
Distributions of breastfeeding support and practices for births in 2015, by state.

The distribution of exclusive breastfeeding at 6 months for infants born in 2015 ranged from 13% in Mississippi to 42.1% in Alaska and breastfeeding at 12 months ranged from 18.3% in Mississippi to 51.7% in Oregon (Fig 1). The number of IBCLCs per 1000 births in 2015 ranged from 1.9 in Washington, DC to 13.4 in Vermont. The number of La Leche League Leaders per 1000 births ranged from 0.2 in Connecticut to 3.3 in Vermont, and the percentage of births in Baby-Friendly Hospitals ranged from 0 in Arkansas, Mississippi, and West Virginia to 47.4 in New Hampshire (Fig 1). Ranges of state values for status of women indices can be found in S1 Table.

All women’s status indices were low or moderately correlated (all Pearson correlation coefficients <0.6), and we retained them in the multivariable models. In bivariate models, political participation, employment and earnings, and poverty were associated with exclusive breastfeeding at 6 months, defined as having a 95% confidence interval (CI) that does not include 1 and a *p*-value < 0.05. The percentage of women in poverty was negatively associated with exclusive breastfeeding at six months (Table 1; IRR = 0.03; 95% CI 0.01, 0.20). Political participation and employment and earnings were positively associated with exclusive breastfeeding at six months. All three breastfeeding supports were positively associated with exclusive breastfeeding at six months (Table 1). All women’s status indices were positively associated with breastfeeding at 12 months, except poverty, which had a negative association (IRR = 0.02; 95% CI 0.00, 0.09). All three breastfeeding supports were positively associated with breastfeeding at 12 months (Table 1).

**Table 1:** Incidence rate ratios estimating bivariate associations between state-level status of women, breastfeeding support, and breastfeeding practices for births in the U.S. in 2015 (n = 51).

	Exclusive breastfeeding at 6 mos.	95% C.I.	Breastfeeding at 12 mos.	95% C.I.
Political participation*	1.02	1.01, 1.03	1.02	1.01, 1.03
Employment and earnings	1.19	1.03, 1.37	1.35	1.08, 1.16
Work and family	1.02	0.95, 1.10	1.06	1.00, 1.11
Poverty among women	0.03	0.01, 0.20	0.02	0.00, 0.09
Reproductive rights	1.02	0.99, 1.05	1.05	1.02, 1.08
IBCLCs per 1000 live births	1.06	1.04, 1.09	1.06	1.03, 1.08
La Leche League Leaders per 1000 live births	1.16	1.10, 1.22	1.15	1.09, 1.22
Baby-Friendly hospital births	1.71	1.04, 2.81	1.67	1.11, 2.51

*Washington, DC does not have a political participation index

In multivariable models controlling for women’s status and the percentages of Black and Hispanic populations, only IBCLCs were positively associated with both outcomes among the breastfeeding supports (Table 2). There was a 5% increase in the rate of exclusive breastfeeding at six months and a 4% increase in the rate of breastfeeding at 12 months with each additional IBCLC per 1000 population, controlling for women’s status. Neither La Leche League leaders nor births in Baby Friendly hospitals had a statistically significant association with either exclusive breastfeeding at six months or breastfeeding at 12 months in multivariable models (Table 2).

**Table 2:** Adjusted incidence rate ratios estimating multivariable associations between state-level breastfeeding support and breastfeeding practices for births in the U.S. in 2015 (n = 50), controlling for women’s status.

	Baby-Friendly hospital births (95% CI)	LLL Leaders per 1000 live births (95% CI)	IBCLCs per 1000 live births (95% CI)
Exclusive breastfeeding at 6 mos.	1.36 (0.83, 2.25)	1.04 (0.96, 1.13)	1.05 (1.03, 1.07)
Breastfeeding at 12 mos.	1.13 (0.84, 1.53)	1.06 (1.00, 1.14)	1.04 (1.02, 1.06)

## Discussion

Lactation is a physiological phase of female reproduction but various socio-economic and other challenges contribute to short breastfeeding durations, as indicated by low breastfeeding prevalence in the United States [7]. Our study suggests that, in states where women have higher status in terms of political participation and employment and earnings, along with a lower proportion living in poverty, women are more likely to be exclusively breastfeeding at 6 months and still breastfeeding at 12 months. In terms of clinical support for breastfeeding, we found individual associations of professional and peer (lay) support along with births in Baby Friendly hospitals with both outcomes. However, neither peer support from La Leche League nor births in Baby Friendly hospitals were associated with either outcome after controlling for women’s status. In contrast, with each additional IBCLC, the rates of both exclusive breastfeeding at 6 months and continued breastfeeding at 12 months increased. This pattern of results would indicate that while both professional and peer support are important resources for individuals [20, 21], only professional support maintains an association with breastfeeding practices measured at the state level when women’s status is controlled for.

Importantly, we found that including women’s status indices in the statistical models attenuated the estimated effects of LLL Leaders and Baby Friendly hospitals on breastfeeding. However, the association between breastfeeding and IBCLC coverage was remarkably resilient to women’s status. A range of studies have found evidence for IBCLC support on breastfeeding practices. One review found a modest increase in breastfeeding to six months with IBCLC support [24]. Many women seek professional support when they have difficulty breastfeeding. Since breastfeeding difficulty is associated with early termination of breastfeeding, the success of IBCLC support may be variable in different situations. Nonetheless, we found a substantial association between the presence of IBCLCs in a state and state breastfeeding practices, controlling for women’s status. Of the three support interventions we examined, IBCLCs provided the most robust association with breastfeeding practices, withstanding the variability of women’s status. States that want to increase breastfeeding rates should consider investing in IBCLC recruiting, training, and deployment, and ensure fair compensation for this professional health service.

Evidence of the effect of La Leche League on breastfeeding practices is limited but there is some evidence for peer and social support. While one review found that social support influenced women’s breastfeeding decisions [20], another found that lay support helped mothers continue to breastfeed where initiation and continued breastfeeding were not high [21]. The bivariate association we observed with breastfeeding practices was not maintained when we controlled for women’s status, perhaps speaking to a limited range of access or influence with different demographic groups. Research indicates that low-income women benefit from peer support [25] but there is limited research on how LLL, as a global organization, supports those populations. More research is needed to understand the association of this peer support mechanism with breastfeeding practices across diverse populations and communities.

Since 1991, the Baby Friendly Hospital Initiative has sought to promote breastfeeding through 10 steps [26]. Hospitals can be certified “Baby Friendly” through a rigorous evaluation process. More than 600 hospitals in the U.S. have been certified “Baby Friendly,” covering almost 30% of births [27]. Evidence supporting the Baby-Friendly Hospital Initiative includes a review which found that the 10 steps—taken together—had a positive impact on breastfeeding practices, with a “dose-response” increase in that the estimated effect was greater with more steps implemented [18]. Another review found an association between the BFHI and breastfeeding initiation and exclusivity in the U.S. but noted that the underlying mechanisms of that association are unclear and more research is needed to investigate BFHI effects in a variety of circumstances, e.g., rural areas and late pre-term infants [28]. Some reviews have pointed to limited evidence supporting individual steps [29]. Finally, other research suggests no association between Baby Friendly Hospital status and statewide breastfeeding initiation rates [19]. Since BFHI offers women support in the early postpartum period, it might be more effective in helping women initiate breastfeeding than in influencing women’s continued breastfeeding behavior at six and 12 months postpartum, although some research found associations between BFHI and longer breastfeeding durations [28].

Notably, there has been some public backlash against the BFHI because some feel that women’s choices are taken away under rules that mandate limited access to breastmilk substitutes (infant formula), no use of pacifiers, and required rooming in of mother and infant [30]. The alternative view, that BFHI adds to women’s choices by ensuring that breastfeeding is at least as well supported as is the use of formula by hospital staff, policies, and facilities, is sometimes lost in the public discourse. With mixed evidence on BFHI’s effectiveness in extending duration outcomes, states and hospitals will want to examine their resource distribution to balance hospital-based with community-based support to reach populations with lower status, e.g., where poverty is higher and employment and earnings are lower.

We controlled for indices of women’s status and each index comprises several indicators. While potentially efficient for statistical modelling purposes, this approach precludes an examination of the relationships of individual indicators with the prevalence of breastfeeding practices. For example, it is notable that aspects of support for working women and their families was not associated with breastfeeding behavior at the population level. There are a few reasons why this might have occurred. First, the IWPR index on work and family combines factors that affect women and families across the lifespan. For example, it incorporates factors affecting the lives of new mothers (e.g., family leave, childcare subsidies) as well as the experiences of potentially older women (elder care). Secondly, there was very little variation in the components of the index that might most affect breastfeeding mothers, specifically family leave availability and paid sick days across the states. Among 50 states and the District of Columbia, only seven states provided some kind of paid leave in 2015 [31]. Thirdly, this index is likely to be internally inconsistent in how its different components might be hypothesized to correlate with breastfeeding. For example, more paid leave and sick days might be reasonably expected to support the duration and exclusivity of women’s breastfeeding behavior; however, in a role as the primary or only breadwinner in the family, a woman’s ability to breastfeed might be hindered, particularly in the absence of paid leave. Future studies may want to take apart this index to capture an indicator of state-wide support for new mothers, specifically.

While this study found that state-wide coverage of IBCLCs was the form of breastfeeding support most strongly associated with breastfeeding practices at the state level, this is not to say that all forms of support (hospital, professional, lay) do not help individual women breastfeed their infants regardless of women’s status where they live. Indeed, several bodies of literature cited in this paper support that notion [21, 28]. However, this analysis indicates that state-level policies to improve breastfeeding practices or to increase support for breastfeeding may need to be evaluated in light of women’s status in that state because, notably, neither peer support nor Baby Friendly hospitals were associated with breastfeeding after controlling for women’s status. As women’s status improves across the country, it will be important to continue to study its effects on women’s health and the potential mediating role it, and different components of it, may play between policies designed to support women and their health outcomes. Women with lower status, or who live in states where women’s status is lower, may need additional targeted breastfeeding support and resources. It is also worth noting that efforts to improve women’s status *per se* can be an effective strategy for improving child health outcomes, including breastfeeding [1].

The “breastfeeding transition” [32]--which sees first breastfeeding decline as women become ‘released’ from the primacy of the maternal role, become more educated and transition to work that takes them away from their home, only to later rise alongside women’s status--has played out across the globe with insufficient attention to its causes and consequences. On the one hand, this transition indicates that increasing women’s status is complementary and not oppositional to children’s health (as found by Koenen et al. [3]; Vanderlinden and Van de Putte [33]). Hence, in addition to considering increases to traditional forms of breastfeeding support, policy makers and advocates may want to consider the importance of the forces shaping women’s status, as documented in the IWPR indices of political participation, reproductive rights, employment and earnings, work and family, and poverty. The problematic aspect of this transition is that we see the emergence of health disparities by race, income, and education whereby more vulnerable women are less likely to initiate breastfeeding or continue it as per public health recommendations [32]. Maternal access to clinical resources, worksite and broader community support are often cited as reasons behind these disparities [8]. More research is needed on the different pathways by which the status of women and gender (in)equality affects breastfeeding practices and how status differs from various associated factors, such as marital status and education [33].

### Limitations

This is an ecological study of associations of coverage for three nationally reported breastfeeding supports with breastfeeding practices, controlling for women’s status. While different study designs (e.g., cohort) could yield more robust evidence, they would be costly and require thoughtful controlling for many individual and environmental factors. This ecological design provides a useful snapshot of associations for state policy makers to consider when planning investment in interventions to support breastfeeding. In addition, and as noted earlier, this design did not permit examination of single components of indices.

## Conclusions

In this study, political participation, poverty, and employment and earnings were associated with state-level breastfeeding practices, and thus, may be important contributors to maternal and child health. While peer, professional, and hospital-based breastfeeding support were associated with state-level breastfeeding rates, only professional support, specifically, the presence of IBCLCs, was substantially associated with state breastfeeding rates after controlling for women’s status. Increasing the number of IBCLCs in each state is one important strategy for improving breastfeeding practices.

## Supporting information

S1 Table Status of women indices, 2013, by state. (DOCX)

S1 Dataset (CSV)
