# Supplementary material for: Women’s status, breastfeeding support, and breastfeeding practices in the United States
Source: PLoS One. 2022 Sep 28;17(9):e0275021. doi: 10.1371/journal.pone.0275021 (PMC9518909; doi:10.1371/journal.pone.0275021)
Supplement: S1 Table — (DOCX) [file pone.0275021.s001.docx]

**S1 Table. Status of Women Indices, 2013, by state***

| State | Political participation | Employment and earnings | Work and family | Poverty among women | Reproductive rights |
| --- | --- | --- | --- | --- | --- |
| Alabama | -4.15 | 3.69 | 3.13 | 0.186 | 1.91 |
| Alaska | -1.75 | 4.32 | 4.34 | 0.09 | 2.83 |
| Arizona | 1.79 | 3.86 | 3.14 | 0.174 | 3.36 |
| Arkansas | -5.93 | 3.58 | 4.63 | 0.184 | 1.72 |
| California | 4.84 | 4.13 | 5.3 | 0.16 | 5.24 |
| Colorado | 0.77 | 4.2 | 4.53 | 0.13 | 3.71 |
| Connecticut | 2.32 | 4.35 | 4.09 | 0.106 | 5.95 |
| Delaware | -1.28 | 4.2 | 3.85 | 0.13 | 3.82 |
| District of Columbia | No value | 5.33 | 5.2 | 0.189 | 5.5 |
| Florida | -0.93 | 3.82 | 2.82 | 0.163 | 2.79 |
| Georgia | -5.18 | 3.94 | 4.19 | 0.183 | 2.85 |
| Hawaii | 1.22 | 4.05 | 4.45 | 0.107 | 6 |
| Idaho | -3.71 | 3.54 | 2.61 | 0.152 | 0.81 |
| Illinois | -0.86 | 4.11 | 3.49 | 0.145 | 4.78 |
| Indiana | 0.17 | 3.76 | 2.03 | 0.159 | 1.89 |
| Iowa | 2.9 | 3.93 | 4.62 | 0.137 | 3.83 |
| Kansas | -1.54 | 3.99 | 3.48 | 0.135 | 0.76 |
| Kentucky | -2.77 | 3.73 | 3.44 | 0.191 | 2.43 |
| Louisiana | -5.55 | 3.56 | 4.11 | 0.2 | 1.62 |
| Maine | 9.09 | 4.03 | 4.57 | 0.13 | 3.53 |
| Maryland | 1.16 | 4.72 | 4.06 | 0.104 | 6.14 |
| Massachusetts | 7.82 | 4.57 | 3.86 | 0.122 | 4.74 |
| Michigan | 1.74 | 3.85 | 2.75 | 0.164 | 1.66 |
| Minnesota | 9.86 | 4.24 | 4.76 | 0.113 | 4.58 |
| Mississippi | -1.21 | 3.57 | 2.55 | 0.243 | 2.09 |
| Missouri | 2.37 | 3.88 | 3.64 | 0.158 | 1.68 |
| Montana | 2.8 | 3.7 | 2.3 | 0.176 | 5.04 |
| Nebraska | -2.81 | 3.87 | 4.16 | 0.129 | 0.59 |
| Nevada | -4.46 | 3.75 | 3.91 | 0.147 | 4.44 |
| New Hampshire | 14.4 | 4.2 | 3.4 | 0.092 | 3.55 |
| New Jersey | -1.49 | 4.39 | 4.99 | 0.111 | 6.08 |
| New Mexico | -0.07 | 3.87 | 3.65 | 0.215 | 5.02 |
| New York | -0.06 | 4.34 | 5.55 | 0.158 | 5.59 |
| North Carolina | 1.59 | 3.97 | 3.35 | 0.174 | 3.01 |
| North Dakota | 0.16 | 3.95 | 2.93 | 0.136 | 2.27 |
| Ohio | -1.21 | 3.89 | 3.27 | 0.153 | 2.99 |
| Oklahoma | -2.76 | 3.78 | 4.5 | 0.167 | 1.95 |
| Oregon | 6.82 | 4 | 4.89 | 0.163 | 6.28 |
| Pennsylvania | -5.29 | 3.97 | 3.43 | 0.135 | 2.53 |
| Rhode Island | -0.14 | 4.24 | 4.94 | 0.138 | 4.63 |
| South Carolina | -3.09 | 3.73 | 3.64 | 0.181 | 3.07 |
| South Dakota | -0.75 | 3.74 | 3.07 | 0.151 | 0.23 |
| Tennessee | -3.01 | 3.86 | 3.03 | 0.175 | 1.42 |
| Texas | -6.22 | 3.87 | 3.34 | 0.168 | 2.19 |
| Utah | -8.12 | 3.76 | 2.27 | 0.135 | 2.16 |
| Vermont | 1.97 | 4.25 | 4.33 | 0.137 | 6.15 |
| Virginia | -4.67 | 4.25 | 2.69 | 0.123 | 2.24 |
| Washington | 8.35 | 4.09 | 4.44 | 0.139 | 5.2 |
| West Virginia | -6.08 | 3.43 | 3.77 | 0.183 | 4.35 |
| Wisconsin | 4.9 | 3.98 | 4.31 | 0.138 | 1.9 |
| Wyoming | -1.97 | 3.91 | 2.6 | 0.109 | 2.21 |

*As reported by Institute of Women’s Policy Research (2020) <https://statusofwomendata.org/>. Accessed 12 July 2020. Political participation, Employment and Earnings, Work and Family, and Reproductive rights are indices combining several indicators; this study uses poverty among women as a single indicator: the percentage of women living in poverty.
